# Effect of Auger recombination on transient optical properties in XUV and soft X-ray irradiated silicon nitride

**DOI:** 10.1038/s41598-021-84677-w

**Published:** 2021-03-04

**Authors:** Victor Tkachenko, Vladimir Lipp, Martin Büscher, Flavio Capotondi, Hauke Höppner, Nikita Medvedev, Emanuele Pedersoli, Mark J. Prandolini, Giulio M. Rossi, Franz Tavella, Sven Toleikis, Matthew Windeler, Beata Ziaja, Ulrich Teubner

**Affiliations:** 1 Institute for Laser and Optics, Hochschule Emden/Leer-University of Applied Sciences, Constantiaplatz 4, 26723 Emden, Germany; 2 Institute of Nuclear Physics, Polish Academy of Sciences, Radzikowskiego 152, 31-342 Kraków, Poland; 3 European XFEL GmbH, Holzkoppel 4, 22869 Schenefeld, Germany; 4 Center for Free-Electron Laser Science CFEL, Deutsches Elektronen-Synchrotron DESY, Notkestrasse 85, 22607 Hamburg, Germany; 5 Elettra-Sincrotrone Trieste S.C.p.A, 34149 Trieste, Basovizza Italy; 6 Institute of Radiation Physics, Helmholtz-Zentrum Dresden-Rossendorf e.V., Bautzner Landstrasse 400, 01328 Dresden, Germany; 7 Institute of Physics CAS, v.v.i., Na Slovance 2, 182 21 Prague, Czech Republic; 8 Institute of Plasma Physics CAS, v.v.i., Za Slovankou 3, 182 00 Prague, Czech Republic; 9 Institut für Experimentalphysik, Universität Hamburg, Luruper Chaussee 149, 22761 Hamburg, Germany; 10 SLAC National Accelerator Laboratory, Menlo Park, CA, 94025 USA; 11 Deutsches Elektronen-Synchrotron DESY, Notkestrasse 85 22607 Hamburg, Germany; 12 Institute of Physics, Carl von Ossietzky University, Carl-von-Ossietzky-Str. 9-11, 26111 Oldenburg, Germany

**Keywords:** Condensed-matter physics, Ultrafast lasers

## Abstract

Spatially encoded measurements of transient optical transmissivity became a standard tool for temporal diagnostics of free-electron-laser (FEL) pulses, as well as for the arrival time measurements in X-ray pump and optical probe experiments. The modern experimental techniques can measure changes in optical coefficients with a temporal resolution better than 10 fs. This, in an ideal case, would imply a similar resolution for the temporal pulse properties and the arrival time jitter between the FEL and optical laser pulses. However, carrier transport within the material and out of its surface, as well as carrier recombination may, in addition, significantly decrease the number of carriers. This would strongly affect the transient optical properties, making the diagnostic measurement inaccurate. Below we analyze in detail the effects of those processes on the optical properties of XUV and soft X-ray irradiated Si${_3}$N$_4$, on sub-picosecond timescales. Si${_3}$N$_4$ is a wide-gap insulating material widely used for FEL pulse diagnostics. Theoretical predictions are compared with the published results of two experiments at FERMI and LCLS facilities, and with our own recent measurement. The comparison indicates that three body Auger recombination strongly affects the optical response of Si${_3}$N$_4$ after its collisional ionization stops. By deconvolving the contribution of Auger recombination, in future applications one could regain a high temporal resolution for the reconstruction of the FEL pulse properties measured with a Si${_3}$N$_4$-based diagnostics tool.

## Introduction

Temporal diagnostics of free-electron laser (FEL) pulses, also applicable for the arrival time jitter measurements in X-ray pump-optical probe experiments, is now widely used at FEL facilities^1–3^. They are based on spatially encoded measurement of transient optical coefficients, currently possible with a temporal resolution better than 10 fs^1,2,4^. In an ideal case, this would also imply a comparable resolution for temporal pulse properties and arrival time jitter between the two pulses. The precision of the measurement depends on the FEL parameters (e.g., stability and spectral content) and optical pulse parameters (e.g., pulse duration). In Ref.^3^ we achieved about 6-15 % accuracy for the typical FERMI settings, see Table 1 in the section ’Method B’ of Ref.^3^. For our experiment presented below, we can assume the same accuracy as the experimental conditions were very similar to those in^3^.

The measurement scheme^1–3^ is based on the encoding of the temporal sample evolution on the spatial coordinate of the FEL irradiated tilted sample. Through the temporal sample evolution we understand here the increase of the free-electron density in the sample within the FEL irradiated area. The non-normal incidence of the incoming FEL beam translates into the spatial encoding of the incoming excitation times which can then be ’read’ by the probe pulse. Originally, this method of the pulse duration measurement was first proposed in^5^ for optical pulses (see there for further details). The idea and the basics of this method was applied to FEL pulses in^1^ and^2^, and later in^3^. The method uses the pump-pulse-induced ultrafast switching of optical refractive indices followed with a common cross-correlation technique.

During a FEL pulse diagnostic measurement, incoming X-rays deposit energy within the material through a photoabsorption process. Photo- and Auger electrons released from the valence band and deeper-lying atomic shells ionize the material further through collisional (impact) ionization. As a consequence, further electrons are released, which form electron cascades. The measured optical parameters (transmissivity, reflectivity and absorption) reflect the changes in the density of excited electrons within the material and can be used as a diagnostic tool sensitive enough to detect even weak electronic excitation. Theoretical modeling is performed with a hybrid simulation tool XTANT. It provides a transient dielectric function and, therefore, allows to predict the corresponding transient optical coefficients.

The application of transient optical properties to measure the FEL pulse duration relies on the fact that X-ray FEL pulse triggers the production of free-electrons in the target. Their density increases until electron impact ionization stops. This occurs when the energies of all excited electrons fall below the material’s ionization threshold, and, therefore, no more electrons (or, in semiconductors/insulators, no more electron-hole pairs) can be excited.

The above scheme typically relies on the following ideal conditions during the optical probing:

(i) no electron/hole escape into deeper parts of the material, or from its surface,
(ii) all excited carriers stay within the lateral overlap of the pump and probe beams,
(iii) no electron/hole recombination processes occurr on the timescales of the measurement.

Under these conditions, the density of carriers probed by the optical beam would accurately follow the (collisional) ionization processes induced by X-ray pulses^6^. However, if any of the processes mentioned in (i)–(iii) occured, that would additionally affect the measured transient optical properties and complicate the interpretation of the measurement. In order to regain access to the information on the increase of the free-electron density due to ionization processes, one would have to deconvolve the contribution of processes (i)–(iii) from the measured transient optical property.

To illustrate, the transient optical reflectivity of Sm$_{0.9}$Y$_{0.1}$S measured in^7^ (Fig. 2b, Fig. 4 in Ref.^7^) and the transmissivity of SiO$_2$ measured in^1^ show the presence of such ’carrier loss’ processes which decrease the number of carriers in the spatial overlap of the pumping and probing beams. This results in a progressing ’recovery’ of the transient optical coefficients towards their equilibrium values.

In contrast, the optical reflectivity of Si${_3}$N$_4$ measured in Ref.^7^ reaches an almost stable value after the ionization processes stop. The material seems, therefore, a very good candidate for a pulse diagnostic tool (see, e.g.,^8–14^). However, it shows a different behaviour in the XUV regime. Fig. 10a in Ref.^3^ shows the transmissivity of Si${_3}$N$_4$ after irradiation with a pulse of $\sim$ 50 eV photon energy and a duration of $\sim$ 75 fs FWHM. After reaching the minimum (corresponding to the collisional ionization stop), the transmissivity curve strongly increases on a 100 fs timescale. This behaviour seems at first glance to be in contrast with the observation by Krupin et al. in^7^, where the corresponding increase was much slower.

**Figure 1:**
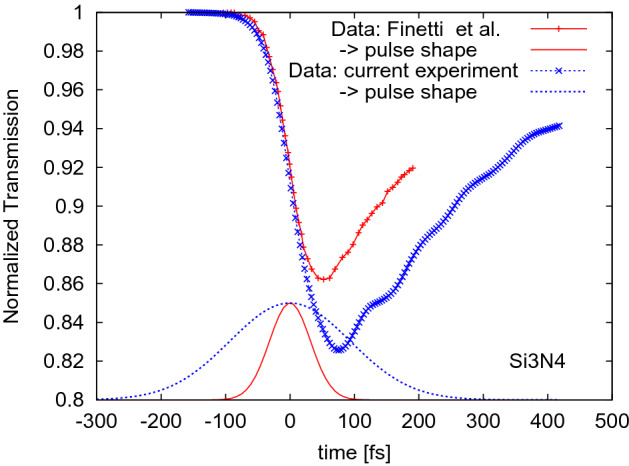
Normalized transmissivity, $T(t)/T_{equib}$ of XUV irradiated Si${_3}$N$_4$ recorded in the experiment performed at the FERMI facility by Finetti et al.^3^ (pulse duration of 75 fs FWHM) and in our current experiment (pulse duration of 220 fs FWHM), when probed with the optical pulses of wavelength $\lambda = 630$ nm. Temporal profiles of XUV pulses are also schematically depicted. The experimental data represent averages over at least 600 single shots.

A further experiment, dedicated to a quantitative study of these diagnostics tools, was performed by our team at the FERMI facility in December 2017. The experimental setup was very similar to the experiment by Finetti et al.^3^. The measurement scheme and the list of the experimental parameters can be found in the section ’Methods’. During our experiment, a 1 $\mu$m-thick silicon nitride layer was irradiated with a pulse of 50 eV photon energy and a duration of 220 fs FWHM. Fig. 1 compares the normalized transmissivities, $T(t)/T_{equib}$ from^3^ and the current experiment, where *T*(*t*) denotes the transient transmissivity. It is divided by $T_{equib}$ which corresponds to the equilibrium transmissivity value before the XUV irradiation. The 12 fs pulse duration of the optical pulse used in our current experiment reveals more details on the optical response. The normalized transmissivity recorded shows a similar behaviour to our earlier experiment (see^3^, cf. Fig. 10a therein). Both curves drop initially to a minimal value, and then start again to increase, with the slopes similar to each other.

The purpose of this study is to investigate physical processes driving the recovery of the transient optical properties on sub-picosecond timescales in silicon nitride irradiated with XUV and soft X-rays. A similar question was addressed in^9^, however, with the analysis focused on *picosecond* timescales and in the XUV irradiation regime. Modified rate equations based on a two-temperature-model were used in^9^ to model the transient optical properties. Global effective ’carrier balance’ coefficients were introduced into those equations to describe the overall charge balance, including ’carrier-gain’ and ’carrier-loss’ processes. Their average values were estimated by fitting the experimental data. With the fitted effective coefficients, the experimental data on Si${_3}$N$_4$ membranes of different thicknesses, excited with the XUV photons of $\sim$ 50 eV energy, could be well parametrized.

Here, we aim at a quantitative understanding of the contributions of carrier-loss processes, in order to identify which of them affects mostly the transient optical properties in silicon nitride on *sub-picosecond* timescales. This study can have strong implications for applications of silicon nitride in the pulse and arrival time diagnostics of femtosecond XUV and X-ray pulses at FEL facilities. The detailed understanding of the behaviour of Si${_3}$N$_4$ can also improve the general understanding of the electronic kinetics in X-ray irradiated materials.

## Methods

Below we present a description of experimental and modeling methods used in our study.

### Experimental set-up and parameters

**Figure 2:**
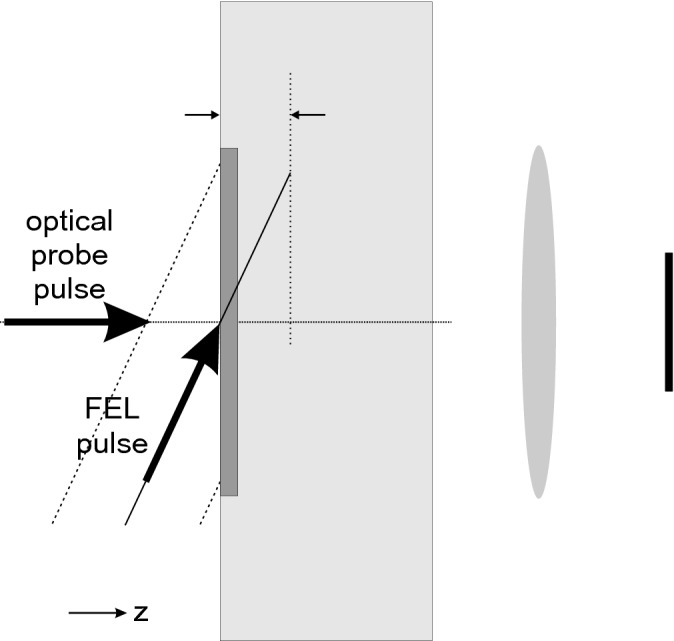
Illustration of the experimental pump-probe scheme. The FEL pump pulse irradiates the tilted sample (light grey box of thickness *d*) at a grazing angle. The extended size of the FEL focus is indicated by the dashed inclined lines. This also sets the temporal window (see, e.g.,^3^). The transmission of the optical pulse is observed by an imaging system (here indicated by a schematic lens and a black box as the detector). The relevant area, where the transmission is observed, is restricted to the FEL-pulse irradiated region. The penetration depth of the FEL pulse is indicated by arrows. For comparison, the thickness of the dark grey box indicates the range of the released photoelectrons.

Both the XUV FEL-pump—optical probe experiments were carried out at FERMI FEL facility. The measurement scheme has been described in detail in Refs.^1–3,15^. It is based on the encoding the temporal evolution within a FEL irradiated, tilted sample on the spatial coordinate of the sample. The temporal pulse shape in both experiments was Gaussian as the FERMI facility uses a seeded FEL. The pulse duration measurement in both experiments was performed with the same scheme described in^3^. Below we list the parameters of the discussed FERMI experiments and also those from the LCLS experiment by Krupin et al.^7^ on Si${_3}$N$_4$, and sketch the experimental scheme (Fig. 2).

- Finetti et al.^3^:
- $F_{FEL}\le$ 0.02 J/cm$^2$—FEL pulse fluence
- $\lambda _{FEL} =$ 26.17 nm—FEL pulse wavelength
- $\tau _{FEL} =$ 74.9 fs—FEL pulse duration
- $\lambda _{probe} =$ 630 nm—probe pulse wavelength
- $\tau _{probe} =$ 30 fs—probe pulse duration
- $S_{probe} =$ 1 mm$^2$—probe pulse spot diameter
- $d =$ 1000 nm—Si${_3}$N$_4$ sample thickness
- $\sigma =$20$^{\circ }$—FEL grazing angle
- $S_{pump} =$ 150 $\mu$m $\times$ 300 $\upmu$m—FEL spot size

- Current experiment:
- $F_{FEL}\le$ 0.02 J/cm$^2$—FEL pulse fluence
- $\lambda _{FEL} =$ 26.3 nm—FEL pulse wavelength
- $\tau _{FEL} =$ 220 fs—FEL pulse duration
- $\lambda _{probe} =$ 630 nm—probe pulse wavelength
- $\tau _{probe} =$ 12 fs—probe pulse duration
- $S_{probe}\sim$ 2 mm$^2$—probe pulse spot size
- $d =$ 1000 nm - Si${_3}$N$_4$ sample thickness
- $\sigma =$20$^{\circ }$—FEL grazing angle
- $S_{pump} =$ 113.4 $\upmu$m $\times$ 9.5 $\upmu$m—FEL spot size

- Krupin et al.^7^:
- $F_{FEL}\le$ 45 mJ/cm$^2$—FEL pulse fluence
- $\lambda _{FEL} =$ 2.3 nm—FEL pulse wavelength
- $\tau _{FEL} =$ 50–150 fs—FEL pulse duration
- $\lambda _{probe} =$ 800 nm—probe pulse wavelength
- $\tau _{probe} =$ 100 fs—probe pulse duration
- $$S_{probe}$$—not specified
- $\sigma =$45$^{\circ }$—FEL grazing angle
- *d*—Si${_3}$N$_4$ sample thickness—not specified in^7^
- *S*—FEL spot size—not specified in^7^

### Simulations of electron propagation in materials with XCascade 3D code

To describe 3D ballistic and diffusive transport of XUV-excited free electrons in Si${_3}$N$_4$, we apply our in-house classical Monte Carlo code, XCascade 3D^16,17^.

In the code, the material is assumed to be a homogeneous bulk of atomic/molecular density corresponding to the density of the modeled material. This allows us to utilize the Poisson distribution for sampling mean free paths of carriers. Atomic cross sections are applied to model the photon absorption and the electron-atom scattering within the material. This approximate treatment is fully justified for high-energy X-rays and for high-energy electron-atom collisions, since they excite electrons from the deeply lying core levels.

The created deep-shell hole can undergo either an Auger or a radiative decay (with the corresponding lifetimes^18^). For light elements, the predominant decay channel is single Auger decay, during which the excess energy from the hole relaxation is used to create another secondary electron (Auger electron). For heavier elements, many-step Auger decays may occur, resulting in several additional valence holes and free electrons.

Released free electrons propagate further in the material. The trajectory of an energetic electron (photoelectron, incident electron, or secondary electron) is modeled as a straight line until an elastic scattering on an atom or an inelastic collision with an atom (here, always with impact ionization) occurs. In our scheme, elastic collisions change only the direction of the propagating electron. During an inelastic collision, the electron, in addition to the changing the direction, loses energy to excite a secondary electron.

**Figure 3:**
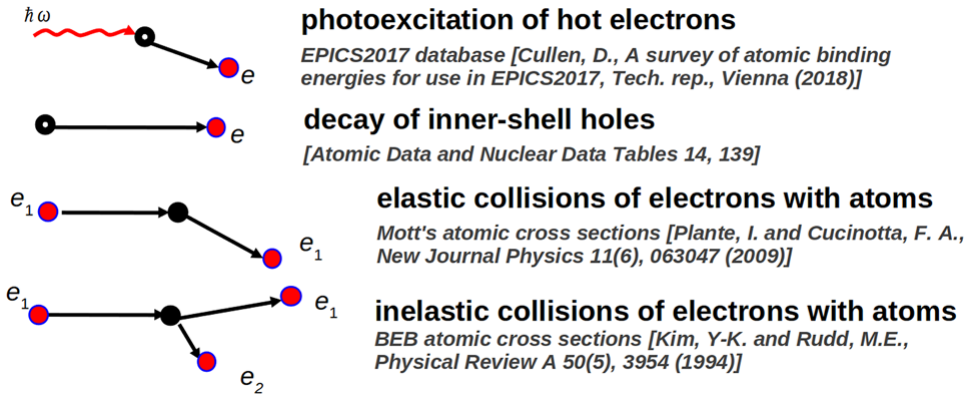
Schematic illustration of X-ray/XUV induced microscopic processes treated by XCascade 3D code.

According to the implemented anisotropic electron scattering scheme^19^, high-energy electrons mostly scatter on atoms in the forward direction. This triggers fast ballistic electron transport. When electron energy decreases below the Hartree energy ($\sim$ 27 eV), the random scattering directions prevail, which results in the following slow diffusive motion of the electron. Each electron propagates until it can no longer trigger an impact ionization, i.e., until its energy decreases below the lowest ionization potential of the target atoms. The code relies on EPICS2017 database of photoabsorption cross sections^20^ and binary-encounter-Bethe cross sections for electron inelastic scattering on atoms^21,22^. The elastic scattering is described with Mott’s elastic cross sections^23^. The atomic ionization potentials are also taken from the EPICS2017 database. Graphical illustration of XUV-induced microscopic processes treated by XCascade 3D is shown in Fig. 3.

Similarly as other transport Monte Carlo codes, the XCascade-3D scheme assumes that the fluence of the X-ray pulse is low. Consequently, one can neglect double excitations of atoms by FEL photons. This also allows to neglect band structure changes and electron-electron correlation effects within the excited electron ensemble: interaction between the excited electrons, atoms, and the Pauli blocking. These assumptions hold under the condition that the maximal electron densities achieved are much lower than the atomic densities of the target, i.e., the average ionization degree is less that 10 %. The target can then be assumed to be undamaged and unexcited. Correspondingly, cross sections and rates for neutral material are used. In addition, all electrons considered are nonrelativistic.

### Estimation of electron transport and Auger recombination coefficients from the experimental transmissivity and reflectivity curves

The evolution of the carrier density in X-ray irradiated sample is affected by the interplay of ’source’ and ’sink’ processes which lead to the density increase and decrease respectively. It can be described by a rate equation (cf. Eq. 4 in^9^) which contains these source and sink terms ( $\Gamma _{e,h}$) for electron and hole carriers:

1 $$\begin{aligned} dn_{e,h}/dt = \Gamma _{e,h}^{phot}+\Gamma _{e,h}^{AD}+ \Gamma _{e,h}^{II} (n_{e,h}) - \Gamma _{e,h} ^{AR}(n_{e,h}) - \Gamma _{e,h} ^{transp}(n_{e,h}). \end{aligned}$$

They describe the change of electron density due to: (i) photoionization, $\Gamma _{e,h}^{phot}$, (ii) emission of electrons after Auger decays of core states, $\Gamma _{e,h}^{AD}$, (iii) impact (collisional) ionization by electrons, $\Gamma _{e,h}^{II} (n_{e,h})$, (iv) Auger recombination, $\Gamma _{e,h} ^{AR}(n_{e,h})$, and (v) electron ballistic and diffusive transport, $\Gamma _{e,h} ^{transp}(n_{e,h})$. The source terms, $\Gamma _{e,h}^{phot (AD)}$ and $\Gamma _{e,h}^{II} (n_{e,h})$ stop to contribute at some time, $t_{max}$, when photoinduced and collisional ionization finish. At this time, the electron density reaches its maximum value.

Using the Drude model (see, e.g.,^24^), the information on transient optical properties can be obtained from the transient electron density. The maximum value of electron density then translates into an extremum of the transmissivity or reflectivity. At $t>t_{max}$, only sink processes contribute to the evolution of the electron density, and this regime is in the focus of our study. The rate equation valid for $t> t_{max}$, to which only sink terms contribute, reads:

2 $$\begin{aligned} dn_{e,h}/dt = - \Gamma _{e,h}^{AR}(n_{e,h}) - \Gamma _{e,h}^{transp}(n_{e,h}). \end{aligned}$$

Both terms depend on the electronic density. The Auger term has a cubic dependence (see the discussion later in the text), and the transport term has a linear dependence on the transient electron density. The final equation takes then the form:

3 $$\begin{aligned} dn_{e,h}/dt = - C_{AR}\cdot n_{e,h}^3 - C_{transp}\cdot n_{e,h}. \end{aligned}$$

with the Auger recombination and transport coefficients, $C_{AR}$ and $C_{transp}$ respectively.

Instead of solving the general rate equation (1), we proceed in the following way. We use a time-dependent term from^24^ to represent the contribution of photoionization, Auger decay and impact ionization processes at $t< t_{max}$. This term contains one free parameter, *a*, which is later fitted (see Supplementary Materials to^24^). In addition, we also include terms with rates corresponding to Auger recombination and electron transport both at $t< t_{max}$ and $t>t_{max}$, as in Eq. (3). The Auger recombination coefficient from Auger recombination term is then another free parameter in the equation, as the electron transport coefficient is already known from the XCASCADE 3D simulations (see the discussion later in the text). We fit the modified Eq. (1) to the data in the whole time regime, $t< t_{max}$ and $t>t_{max}$. The values of $C_{AR}$ (and parameter *a*) are then obtained.

## Electron transport and Auger recombination in X-ray irradiated silicon nitride

Earlier detailed studies (see^25–28^ and references therein) showed that high-energy electrons predominantly scatter on atoms in the forward direction, which triggers their fast ballistic transport. If, due to the on-going impact ionizations, the kinetic energies of the carriers decrease below $\sim$ 27 eV, they start to scatter isotropically. This initiates their diffusive transport (which is much slower than the ballistic transport) towards the deeper layers of the material. The transport processes can cause local decrease of the carrier density. This would be detected as the recovery of the measured optical parameters towards their equilibrium values.

Already a rough comparison of the spatial scales of photon and electron attenuation in Si${_3}$N$_4$, allows one to expect that the transport of the electrons and holes in the material cannot be a predominant channel of the ’carrier loss’ leading to the recovery of the optical parameters towards equilibrium on timescales less than 1 ps. The (ballistic) range for electrons, $R_{max,el}$ released in Si${_3}$N$_4$ by an impact of 50 eV photons equals only 1.7 nm, according to our XCASCADE-3D simulations. The electron range is defined as the maximal distance which such an electron can travel in the material before its kinetic energy becames so low that it cannot ionize more electrons. Let us compare it with the spatial scale of photon attenuation. The photon penetration depth, $\lambda _{P}$, is defined as the depth at which the intensity of the radiation inside the material falls to 1/*e* of its initial value. The distance $R_{3\, \lambda _{P}}=3 \cdot \lambda _{P}$ corresponds to a material depth at which $\sim 95$% of the energy carried by a photon beam was absorbed in the material. For a pulse with 50 eV photons, arriving at the grazing angle of 20$^{\circ }$ (i.e., the considered experimental geometry), the photon penetration depth is $\sim$ 6 nm. When one compares $R_{max,el}$ with $R_{3\, \lambda _{P}}$, this yields the ratio, $R_{max,el}\,/R_{3\, \lambda _{P}}\sim 0.1$. It becomes then clear that the majority of the released electrons will remain within the material depth down to $R_{3\, \lambda _{P}}$, as $R_{max,el}\,<< R_{3\, \lambda _{P}}$.

To verify this expectation, with the XCascade 3D code we calculated the distribution of electrons and holes as a function of time, *t*, and depth into the material, *z*, in an XUV irradiated 1 $\mu$m-thick layer of Si${_3}$N$_4$. Lateral carrier distribution in (*x*, *y*) plane was assumed to be uniform for a fixed *z*, due to a large lateral overlap of the pump and probe beams (see ’Methods’). This large overlap also made any lateral electron escape negligible in this case. During the simulations, both electrons and holes propagated in the material. However, the holes, due to their low kinetic energy, scattered only elastically on the atoms. Along the *z* direction, the material layer was divided into a set of sublayers. In each sublayer the transient electron density was calculated. In this way, we accounted for the gradient of electron density in *z* and the respective change of the refractive index along the optical path.

For a weak electronic excitation, much below the structural damage threshold, as that considered here, optical coefficients can be calculated in the framework of the well-known Drude model^29,30^. Using the Drude model, material’s transient optical properties can be evaluated from the predicted transient carrier density, taking into account both the respective electron and hole contributions, as described, e.g., in^24,31^. In such way the transmissivity was here calculated, using the graded optical medium approach from^30^, applied also in^24^. Let us emphasize that surface electron escape was accounted for by our model, similarly as in^32^.

The calculations with the XCascade 3D provided us with the transport coefficient, $C_{transp}$. As next, we analyzed the effect of the Auger recombination on the transient optical properties. During the non-radiative three-body process, an electron-hole pair recombines, delivering the excess energy to a third carrier: electron or hole^29^. The respective Auger recombination rates, $\Gamma _{AR}$, are related to carrier concentrations as

4 $$\begin{aligned} \Gamma _{AR,eeh} = C_{eeh}\,n^2 p \end{aligned}$$

or

5 $$\begin{aligned} \Gamma _{AR,ehh} = C_{ehh}\,p^2 n, \end{aligned}$$

where *n* and *p* are the respective electron and hole concentrations. Note that in insulators, $n = p$. The total rate of Auger recombination can then be written as:

6 $$\begin{aligned} \Gamma _{AR}=C_{AR} \cdot n^3, \end{aligned}$$

with the Auger coefficient^33^

7 $$\begin{aligned} C_{AR}=C_{eeh} + C_{ehh}. \end{aligned}$$

For example, for undoped Si, the respective total Auger coefficient is $C_{AR} \approx 3.9\cdot 10^{-31}$ cm$^6$/s at 300 K^34^. The literature does not provide data on the Auger recombination coefficient for Si${_3}$N$_4$.

In our experiment, Si${_3}$N${_4}$ was excited by XUV photons, producing an equal number of electron-hole pairs. As Si${_3}$N${_4}$ is an insulator, the expected Auger recombination scenario is then similar to that in an equilibrium intrinsic semiconductor with the non-doping condition, $n = p$.

Taking into account both: (i) the ballistic and the diffusive transport of carriers (with rates estimated from the XCascade 3D simulations), and (ii) the Auger recombination, we calculated the transient carrier densities within X-ray irradiated Si${_3}$N$_4$ for both experiments performed at the FERMI facility: the previous one^3^, and for our current experiment. With the Drude model, we calculated the respective transmissivity curves (for further details see the section “Methods”).

**Figure 4:**
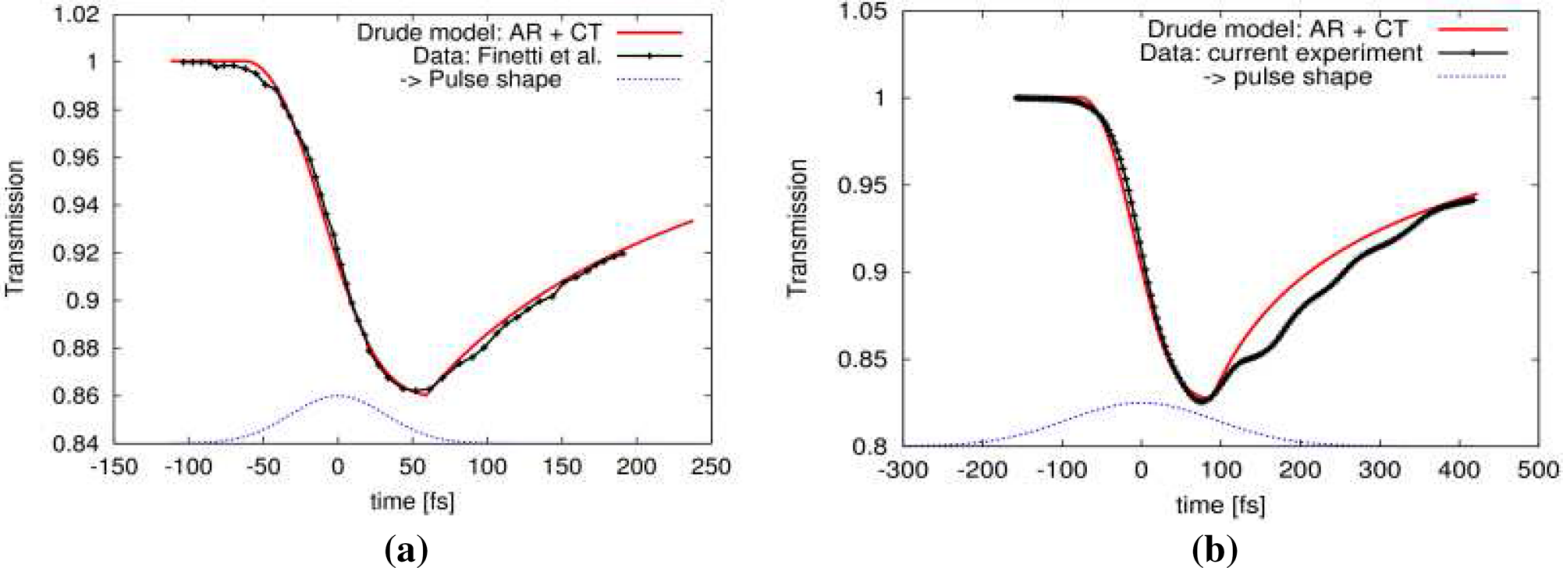
Normalized transmissivity, $T(t)/T_{equib}$ of XUV irradiated Si${_3}$N$_4$ recorded in the experiment performed at: **(a)** the FERMI facility by Finetti et al.^3^ (pulse duration of $\sim$ 75 fs FWHM), and **(b)** in our current experiment (pulse duration of $\sim$ 220 fs FWHM), when probed with the optical pulses of wavelength $\lambda = 630$ nm. The experimental data represent averages over at least 600 single shots. They are compared with the results of the simulation in case when the Auger recombination (AR) and carrier transport (CT) were taken into account. Temporal profile of XUV pulses is also schematically depicted.

**Figure 5:**
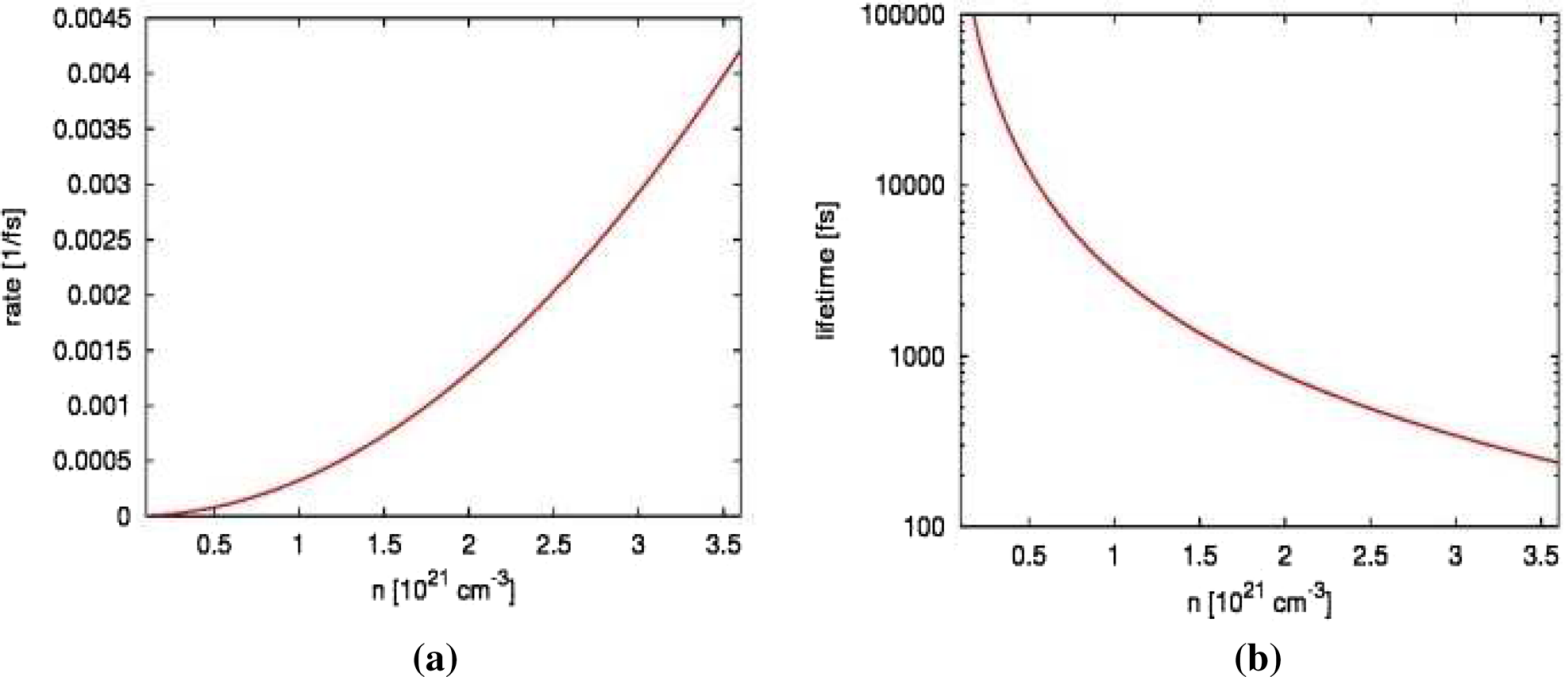
(**a**) The effective recombination rate, $C_{AR}\cdot n^2$, and (**b**) the respective lifetime, $\tau _{AR}=1/(C_{AR}\cdot n^2)$ for $C_{AR}\approx 3.25\cdot 10^{-31}$ cm$^6$/s plotted as a function of free electron density, *n* within the regime of *n* relevant for this study.

Figure  4a,b show the results. After the transmissivity curve reaches its minimum, its recovery towards the equilibrium value is due to the on-going Auger recombination and carrier transport. The effective transport escape rates obtained from the simulations with XCASCADE-3D code were respectively $2.92\cdot 10^{12}$ 1/s for the case (a), and $2.00\cdot 10^{12}$ 1/s for the case (b). The fitted Auger coefficient was found to be, $C_{AR}\approx 3.25\cdot 10^{-31}$ cm$^6$/s, i.e., comparable with that one for undoped silicon. Figure 5a,b below shows the the effective recombination rate, $C_{AR}\cdot n^2$ and the respective lifetime $\tau _{AR}=1/(C_{AR}\cdot n^2)$ as a function of free electron density, *n*, within the regime relevant for this study. Here, the Auger recombination is a slow process, with $\sim$237 fs lifetime for the highest electron density of $3.6\cdot 10^{-21}\,\mathrm{cm}^{-3}$ shown in the plot. This density corresponds to the highest electron density which could be excited in the current experiment. It was estimated from the total photoabsorbed energy. For comparison, the number density of Si${_3}$N$_4$ is $9.53 \cdot 10^{22}$ atoms per cm$^{3}$.

The contributions of transport and recombination processes can be roughly compared after multiplying the Auger coefficient with the carrier density squared, and comparing this value with the effective transport escape rate. The ratio of these coefficients is $\sim$ 1. Thus, we can conclude that in this case carrier transport and Auger recombination contribute equally to the rise of the transmissivity observed experimentally.

**Figure 6:**
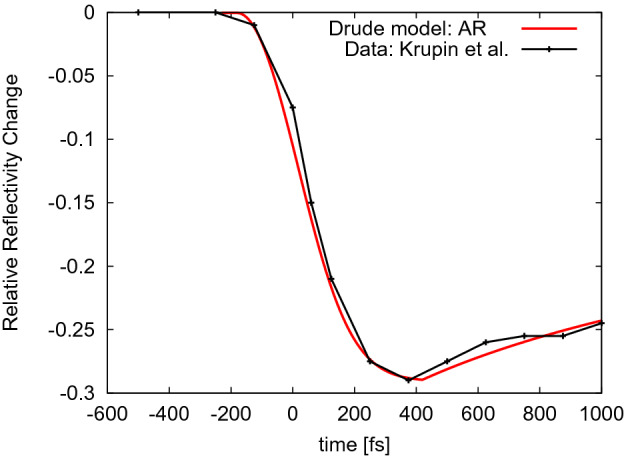
Relative change of reflectivity, $(R(t)-R_{equib}\,) / R_{equib}$ in soft X-ray irradiated Si${_3}$N$_4$ recorded in the experiment by Krupin et al.^7^ (Fig. 3 therein). The data were averaged over many single shots. FEL photon energy was 540 eV. Note that the XFEL pulse duration ($\sim$ 50–150 fs FWHM) was not precisely measured in this experiment. Also, the time ’zero’ denotes here the time instant when the reflectivity starts to decrease. The probe pulse wavelength was $\lambda = 800$ nm. The published data reproduced from Ref. [8] are then compared to the results of our calculations in which only the Auger recombination (AR) was taken into account.

Let us now compare the XUV irradiation regime with the soft X-ray regime. Our calculations with the XCascade 3D code^16^ show that the ranges of electrons released by soft X-ray photons increase, when compared with the XUV case. For example, for the photoelectrons released by 540 eV photons, $R_{max,el}=4.3$ nm, and for the photoelectrons released by 1200 eV photons, it is 17.4 nm. However, at the same time, the photon penetration depth significantly increases, reaching (sub)micron spatial scales. As a result, the relation, $R_{3\, \lambda _{P}}>> R_{max,el}$ is maintained. In particular, for the conditions of the experiment by Krupin et al.^7^, the respective photon penetration depths at the FEL incidence angle of 45$^o$ are 0.2 and 1.6 $\upmu$m respectively. This yields $R_{3\, \lambda _{P}}$ of 600 nm for 540 eV photons and of 4800 nm for 1200 eV photons. The corresponding ratios, $R_{max,el}\, /R_{3\, \lambda _{P}}$ become $7\cdot 10^{-3}$ and $4\cdot 10^{-3}$ respectively. Thus, on the considered *sub-picosecond* timescales, one can then neglect a contribution of carrier transport (including diffusion) to the optical properties. Fig. 6 shows the relative change of the optical reflectivity recorded by Krupin et al. (Fig. 3 in^7^). As mentioned earlier, after the minimum of the reflectivity curve, we observe its further increase. However, it is much slower than that one observed in^3^ and in our current experiment. For confirmation, we applied the Drude model to describe Krupin’s data obtained with pulses of 540 eV photon energy, assuming the effective diffusion coefficient to be zero. The fitted Auger coefficient is, $C_{AR}=3.25\cdot 10^{-31}$ cm$^6$/s, i.e., it is equal to that one obtained in XUV irradiation case. Krupin’s results obtained for photons of higher energies (up to 2000 eV) show even slower recovery of the reflectivity (see Fig. 3 therein), supporting our argument.

Let us comment here on the oscillations of the transmission observed at sub-picosecond times in Fig. 4a,b. Our first general comment is that the better visibility of the oscillations in Fig. 4b when compared to Fig. 4a is due to the much shorter optical probe pulse duration, namely 12 fs instead of 30 fs used in^3^. This determines the differing temporal resolution. To compare, the optical pulse duration in^7^ was around 100 fs, i.e., the resolution time window there was much broader than in the two other cases—too low to detect the fast oscillations. It is difficult to state anything quantitative about the oscillations by analyzing the data with different temporal resolutions. Even the oscillation magnitudes cannot be meaningfully compared. These late-time oscillations could be associated with the increasing temperature of the atomic lattice, due to the progressing energy exchange between electronic and atomic systems, and the resulting stronger atomic vibrations. For a quantitative explanation of this interesting feature, a dedicated experimental study at a high temporal resolution of the optical probe would be necessary. However, this is beyond the scope of the current paper.

Finally, let us mention that our measurements were carried out only at fluences $< 0.02$ J/cm$^2$. No measurements for other fluences were performed. A higher fluence would produce a higher number of free carriers which, in turn, would increase the Auger recombination rate as follows from Eq. (7) and Fig. 5a, and, consequently, lower the Auger recombination lifetime (Fig. 5b). However, at the same time, the minimal value of transmissivity would decrease at higher fluences, due to the higher number of free carriers in the sample. These competing processes would both affect the recovery time for the transmissivity. Therefore, it is difficult to predict the trend without dedicated simulations. This would be an interesting topic for a joint experimental and theoretical study in future.

## Summary and conclusions

Modern experimental techniques enable the measurement of spatially encoded optical coefficients with a temporal resolution below 10 fs. Timing tools, based on those measurements, are applied for XUV and X-ray FEL pulses to determine their temporal characteristics, in particular, their pulse duration and arrival time. Consequently, a comparable diagnostics resolution should be expected. However, the carrier transport outside the lateral spatial overlap of the FEL and the optical probe beams, or from the material surface, as well as carrier recombination, may strongly affect the optical properties already on femtosecond timescales, and make the diagnostic measurement inaccurate. Here we studied in detail the effect of those processes on optical properties of the XUV and soft X-ray irradiated Si${_3}$N$_4$ on subpicosecond timescales.

We found that the carrier transport and Auger recombination had a comparable effect on the transient optical properties of Si${_3}$N$_4$ after the XUV irradiation. The contribution of the transport processes became much smaller in the soft X-ray regime because the ratio between photoelectron range and photon penetration depth strongly decreased due to the increased X-ray photon energy and X-ray grazing angle. Generally, the effect of the carrier transport can be controlled in experiments by choosing such X-ray photon energy and experimental geometry (X-ray grazing angle) which yield a small ratio of $R_{max, el}$ to $R_{3\, \lambda _{P}}$.

On the other hand, we have found that the three-body Auger recombination had a strong effect on the optical response of Si${_3}$N$_4$ in both XUV and soft X-ray irradiation regimes. Therefore, one can expect to regain a high resolution of the temporal FEL pulse properties at a suitably adjusted experimental geometry by deconvolving the Auger recombination contribution from the measured transient optical properties. This conclusion is important for future pulse diagnostic applications at XUV and X-ray FEL facilities, and can increase their accuracy. In particular, in the context of the planned delivery of sub-femtosecond pulses planned at the FEL facilities, e.g., at the Linac Coherent Light Source (LCLS)^35^, achieving and controlling high temporal resolution for pulse diagnostics measurements becomes an absolute necessity.

Note that our results were obtained for low-intensity regime of X-ray and optical lasers, and therefore, cannot be directly linked to the high-intensity laser experiments. However, the Auger recombination will also play a role in the relaxation of high-intensity laser-excited targets.
